# A probabilistic disease progression modeling approach and its application to integrated Huntington’s disease observational data

**DOI:** 10.1093/jamiaopen/ooy060

**Published:** 2019-01-07

**Authors:** Zhaonan Sun, Soumya Ghosh, Ying Li, Yu Cheng, Amrita Mohan, Cristina Sampaio, Jianying Hu

**Affiliations:** 1 Center for Computational Health, IBM T. J. Watson Research Center, 1101 Route 134 Kitchawan Rd, Yorktown Heights, New York 10598, USA; 2 CHDI Management/CHDI Foundation, 155 Village Boulevard, Suite 200, Princeton, New Jersey 08540, USA

**Keywords:** disease progression modeling, continuous-time hidden Markov models, observational study, Huntington’s disease

## Abstract

**Objective:**

Chronic diseases often have long durations with slow, nonlinear progression and complex, and multifaceted manifestation. Modeling the progression of chronic diseases based on observational studies is challenging. We developed a framework to address these challenges by building probabilistic disease progression models to enable better understanding of chronic diseases and provide insights that could lead to better disease management.

**Materials and Methods:**

We developed a framework to build probabilistic disease progression models using observational medical data. The framework consists of two steps. The first step determines the number of disease states. The second step builds a probabilistic disease progression model with the determined number of states. The model discovers typical states along the trajectory of the target disease, learns the characteristics of these states, and transition probabilities between the states. We applied the framework to an integrated observational HD dataset curated from four recent observational HD studies.

**Results:**

The resulting HD progression model identified nine disease states. Compared to state-of-art HD staging system, the model 1) covers wider range of HD progression; 2) is able to quantitatively describe complex changes around the time of clinical diagnosis; 3) discovers multiple potential HD progression pathways; and 4) reveals expected time durations of the identified states.

**Discussion and Conclusion:**

The proposed framework addresses practical challenges in observational data and can help enhance the understanding of progression of chronic diseases. The framework could be applied to other chronic diseases with the help of clinical knowledge.

## INTRODUCTION

Chronic diseases that progress slowly are among the most common, expensive, and debilitating of all health problems.^1^ Modeling symptom progression of chronic diseases enables better understanding of disease prognosis and provides insights into staging systems, which could assist early diagnosis and personalized care, and provide help in the development and evaluation of interventions.

*Disease Progression Modeling* (DPM)^2^ describes the time course of disease status and tracks disease severity over time. Longitudinal information collected in observational studies such as disease registries^3^ and Electronic Health Records^4^ provides rich structured information for data-driven approaches. However, several challenges make the use of such real-world evidence for tracking disease progression difficult. First, although the underlying disease progression processes likely change continuously, observations are only available at discrete, often irregular, time points. Second, an individual patient’s records typically cover only a fraction of the entire progression trajectory, and a comprehensive trajectory must be inferred by stitching together records from a large number of patients, which may not be readily aligned. Third, progression of chronic conditions is usually manifested through multifaceted symptoms. Modeling the complex progression patterns of multiple symptoms is of great value for improving the understanding of a target disease. Last but not least, while a few well studied chronic conditions, such as chronic kidney disease, have widely accepted biomarkers for tracking their natural progression pathways, such knowledge is not available for other less studied conditions, especially rare diseases such as the Huntington’s Disease (HD).

HD is a neurodegenerative disorder caused by an unstable expansion in a trinucleotide (CAG) repeat in the huntingtin (HTT) gene,^5^ and is clinically characterized by the progressive decay of motor and cognitive abilities accompanied by functional and behavioral changes.^6^ Due to its monogenic nature, predictive genetic testing can determine whether an individual will develop the disease. Among genetically confirmed Huntington’s Disease Gene Expansion Carriers (HDGECs), a clinical diagnosis of HD is typically made when an individual exhibits overt, otherwise unexplained extrapyramidal movement disorder. The periods before and after the motor diagnosis are referred to as the premanifest and manifest periods, respectively.

Previous clinical studies on the natural history of HD mainly focus on the motor onset. Stine *et al.* and Duyao *et al.*^7^^,^^8^ identified strong correlation between the age of motor onset and the CAG repeat length. Langbehn *et al.*^9^ developed a parametric survival model based on CAG repeat length to predict the probability of motor onset. Dorsey *et al.*^10^ studied the longitudinal changes of clinical features among HDGECs, and compared with controls. Warner and Sampaio^11^ presented a general class of models, and fitted the models to a selection of structural imaging markers. Despite the increasing understanding of HD progression, several limitations exist in previous studies. First, while motor impairment has been the primary focus in HD clinical studies, cognitive,^12^ and certain behavioral disorders^13^ are also known to surface years before motor onset. Second, most previous studies model one clinical assessment at a time. The multifaceted nature of HD progression calls for a comprehensive characterization of the processes involved.

Recently, several large-scale observational studies have been conducted in HDGECs to better understand the natural history of HD. These studies generated longitudinal datasets from relatively large HDGECs cohorts, providing unprecedented opportunity to investigate the progression of HD.

In this study, we propose a framework based on the Continuous-Time Hidden Markov Model (CTHMM) to address the aforementioned challenges for building disease progression models from observational data. The framework consists of two steps. The first step determines the number of disease states using a grid-search approach. The second step builds a probabilistic disease progression model with the determined number of states. The proposed framework was applied to an integrated HD observational data, and the results are discussed.

### Objective

The aim of this study is to develop a method to address the challenges and build probabilistic disease progression models to enable better understanding of chronic diseases and provide insights for staging systems.

## METHODS

### Continuous-time hidden Markov model

The CTHMM model assumes that the progression of the target disease can be segmented into M distinct disease states, where each disease state captures a typical disease status along its natural course. The underlying progression process of the target disease is assumed to evolve according to a continuous-time Markov process, which is denoted as S(τ), and is parameterized by an M×M transition generator matrix Q, and an M×1initial state probability vector π. The (i,j)-th element of Q, denoted as $Q_{\left(i,j\right)}$, characterizes the intensity of instantaneous transition from disease state i to disease state j, for i≠j. The ith diagonal element $Q_{\left(i,i\right)}=-\sum_{j\neq i}Q_{\left(i,j\right)}$, and the row sums of Q equal to 0. The progression of the target disease is reflected in the transition of disease states. Note that an element $Q_{\left(i,j\right)}=0$ (for i≠j) indicates that patients in disease state i cannot progress into disease state j at an instantaneous time. Different types of disease progression can be specified by imposing various constraints on the structure of Q. For example, a Q with all elements not equal to 0 indicates that a patient in any disease state can progress/recover to any other state. The corresponding model is referred to as the full progression model. A Q with all the lower triangular elements equal to 0 indicates that a disease can only get worse and the progression cannot be reversed. The corresponding model is referred to as the forward progression model. A Q with only the diagonal line and the first L upper off-diagonal lines not equal to 0 indicates that the disease can only progress to the next L states at any instantaneous time. The corresponding model is referred to as the L-th order forward-chain progression model. For disease progression, the most appropriate type of the model (ie, constraints on Q) is specified based on existing knowledge of the target disease. Given Q, the transition probabilities with a time span δ can be calculated by Equation 1 in Wang *et al*.:^4^

(1) $$A_{i,j}\left(\delta \right)=\;{expm(\delta Q)}_{i,j}$$

Although the underlying progression is assumed to be continuous-time, we only observe manifestations of disease states at discrete times. Assume there are N patients in the dataset. Patient n has $T_{n}$ observations, with time stamps $\tau_{1},\ldots ,\tau_{T_{n}}$. Let $Z_{k}$ denote the k-th feature with k=1,…,K, $S_{n,t}$ denote the disease state of patient n at $\tau_{t}$, and $S_{n}=\left\{S_{n,1},\ldots ,S_{n,T_{n}}\right\}$ denote the disease state sequence of the patient. Without loss of generality, we assume that the features under each state follow independent Gaussian distributions, that is, $Z_{n,t,k}|S_{n,t}=m∼N\left(\mu_{m,k},\sigma_{m,k}^{2}\right)$, where $Z_{n,t,k}$ denotes the value of the kth feature of patient n at his tth observation, $\mu_{m,k}$ and $\sigma_{m,k}^{2}$ are the mean and variance of the kth feature under state m. We use $\text{Θ}=\left\{Q,\pi ,\vec{\mu },{\vec{\sigma }}^{2}\right\}$ to denote the collection of parameters in the CTHMM model. Note that the states S are not directly observed. The goal is to estimate Θ and S simultaneously.

The Expectation-Maximization (EM) algorithm [15] was used to estimate the parameters. Specifically, the complete likelihood can be written as follows:

(2) $$P(Z,S,S(\tau );\text{Θ})=\prod_{N}^{n=1}\left\{P(S_{n,1})\prod_{T_{n}}^{t=1}P(S_{n,t}|S_{n,t-1}\right)\prod_{T_{n}}^{t=0}\prod_{K}^{k=1}P(Z_{n,t,k}|S_{n,t})\}$$

The conditional expectation term $E_{P(S,S(\tau )|Z;\Theta )}\left[\log P\left(Z,S,S\left(\tau \right);\Theta \right)\right]$ can be broken down to two terms:

(3) $$E_{P\left(S|Z;\Theta^{'}\right)}\left[log\pi +logP(Z|S)\right]+E_{P\left(S,S(\tau )|Z;\Theta^{'}\right)}\left[logP(S,S(\tau );\Theta )\right]$$

where $\Theta^{'}$ is the value of the parameters from the previous EM iteration. Following equation (II.7) in Metzner *et al.*,^16^ the second term in (4) can be calculated as follows:

(4) EPS,S(τ)|X;Θ'logPS,Sτ;Θ=∑δi,j∈[M]Cij(δ)∑k,l∈M;k≠llog⁡QklENklδ|S;Q'-QklERkδS;Q')

where $C_{ij}(\delta )$ denotes the number of transitions such that $S_{t-1}=i$, $S_{t}=j$, and $\tau_{t}-\tau_{t-1}=\delta$, $N_{kl}(\delta )$ is the number of transitions from state k to state l during time interval δ, and $R_{k}(\delta )$ is the total time the Markov process spends in state k during the time interval δ. In the M-step, we update the transition generator matrix Q and initial probability π as follows,

(5) $$Q_{ij}=\frac{\sum_{\delta ;k,l\in [M]}E\left[N_{ij}\left(\delta \right)\right|S\left(\delta \right)=l,S\left(0\right)=k;Q^{'}]C_{kl}(\delta )}{\sum_{\delta ;k,l\in [M]}E\left[R_{i}\left(\delta \right)\right|S\left(\delta \right)=l,S\left(0\right)=k;Q^{'}]C_{kl}(\delta )},$$

(6) $$\pi_{i}=\frac{\sum_{n=1}^{N}P(S_{n,0}=i;\pi^{'},Q')}{\sum_{n,j}P(S_{n,0}=j;\pi^{'},Q')},$$

where [M] denote the set of integers from 1 to M.

We follow Section 4.2 of Wang et al.^14^ to calculate the two expectation terms $E(N_{kl}(\delta ))$ and $E(R_{k}(\delta ))$, and use the standard forward-backward algorithm to calculate the posterior distributions in (7).

In the E-step, state sequences are updated by the Viterbi algorithm. Next, parameters in the observational model can be updated. Specifically, under the independent Gaussian model, the parameters (μ,σ) can be updated by the sufficient statistics expressed as follows:

(7) $$\mu_{m,k}=\frac{\sum_{n,t}P(S_{n,t}=m)Z_{n,t,k}}{\sum_{n,t}P(S_{n,t}=m)}$$

(8) $$\sigma_{m,k}^{2}=\frac{\sum_{n,t}P(S_{n,t}=m){\left(Z_{n,t,k}-\mu_{m,k}\right)}^{2}}{\sum_{n,t}P(S_{n,t}=m)}$$

Replacing the independent Gaussian model with other (multivariate) distributions in the exponential family is straightforward and would not increase the complexity the algorithm.

### Determine number of disease states M

The CTHMM model assumes that the number of disease states M is predetermined. For some of the most studied chronic diseases which have widely accepted staging systems or biomarkers, Mcan be determined based on clinical knowledge. However, such knowledge is not available for other diseases, especially those rare and less understood diseases such as HD. We used a data-driven approach for determining M for these diseases. To select the optimal M, the dataset is split into a training set and a testing set. First, a series of CTHMM model with various value of M are built on the training set. Each model is then applied to the test set to calculate the fitness measure defined as log-likelihood on the test set given the model. The model with the highest log-likelihood provides the best fit for the data, and its corresponding M is chosen as the optimal M.

### Individual state sequences

Individual state sequences can be obtained using the standard Viterbi algorithm. Furthermore, predictions of future disease states and feature values can be made by leveraging intermediate results from the model. Due to limited space, the detailed description of the method is given Sections S3 and S4 in the Supplementary Material.

## DATASET

The dataset used in this study was integrated from four large-scale prospective observational studies of HD, which are named Enroll-HD,^17^ REGISTRY,^18^ TRACK-HD/TRACK-ON,^13^^,^^19^ and PREDICT-HD,^20^ respectively. In each of the four studies, participants went through annual study visits and generated a diverse set of clinical assessments that span a spectrum of clinical symptoms and manifestations expressed by HD patients. The integrated dataset contains 55782 observations from 16653 HDGECs and 2716 control participants, with the average number of observations (ie, number of study visits) per participant being 2.9. Details about data from the four studies are summarized in Supplementary Material S1, and the integration steps are presented in the Supplementary Material S2. In the rest of this article, we refer to this data as the *integrated HD data*.

Several challenges prohibited directly applying the framework to the integrated HD data. First, clinical assessments may have limited availability due to missing values. Second, not all assessments collected in observational studies were relevant for tracking HD progression. Third, the high-dimensional clinical assessments are essentially manifestations of an unobserved lower-dimensional disease process. Extracting sensitive and efficient representations of the unobserved and heterogeneous progression processes is crucial for the success of DPM.

To address these issues, we exploited the Bayesian Latent Variable Analysis by Ghosh *et al.*^21^ to extract latent factor scores to represent the underlying progression trajectories. Specifically, we extracted the leading three latent factors from each of motor, functional, and cognitive domains. We kept the number of factors equal in the three domains so that we did not bias the final HD progression model towards symptoms in any single domain. The extracted latent factors were concatenated and used as observed features (Z) in the CTHMM model.

## RESULTS

The integrated HD data consists of participants with the number of clinical visits ranging from 1 to 25 (Supplementary Figure S2). Since longitudinal information is essential for DPM, we excluded study visits with missing values and patients with only one clinical visit. We used 3126 HDGECs with at least 4 visits to determine the number of disease states (M), and 8452 HDGECs with at least 2 observations to build the final HD progression model.

It is known that HD symptoms progress slowly over a long period of time, and no known treatment have been demonstrated to be effective in reversing or slowing down its progression. Based on the understandings of HD, we set the HD progression model to be a second-order forward-chain progression model. That is, at any instantaneous time, a patient at state i can have three possibilities: 1) stay at state i; 2) jump to state i+1; or 3) jump to state i+2. In addition, the last disease state was set as an absorbing state.

### Determine the number of disease states

**Figure 1.**
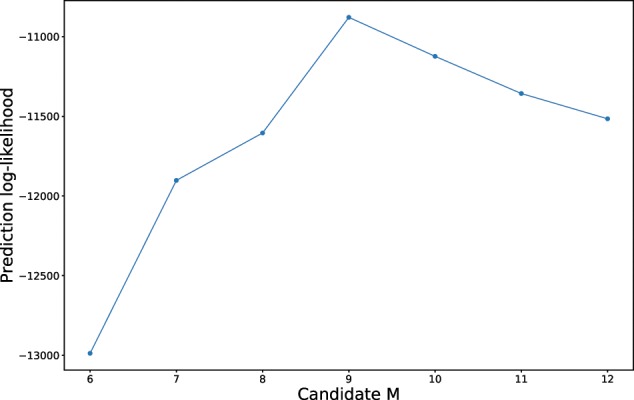
Number of disease states versus prediction log-likelihood.

We randomly split the 3126 participants into a training set (80%) and a testing set (20%). Using the approach described in the Methods section, we trained separate CTHMM models with *M* ranging from 6 to 12, and applied the models on the testing set to calculate the log-likelihood. Figure 1 shows the log-likelihood versus M. The model with 9 states yielded the highest log-likelihood. Therefore, we built the final HD progression model with 9 states.

### Integrated HD progression model

The final HD progression model is referred to as Integrated Huntington’s Disease Progression Model (IHDPM), and we compare it to the Shoulson and Fahn HD stages.^22^ The Shoulson and Fahn stages are defined for patients who have reached their motor onset. The stages are defined based on the Total Functional Capacity, which is a functional score in the UHDRS.

**Figure 2.**
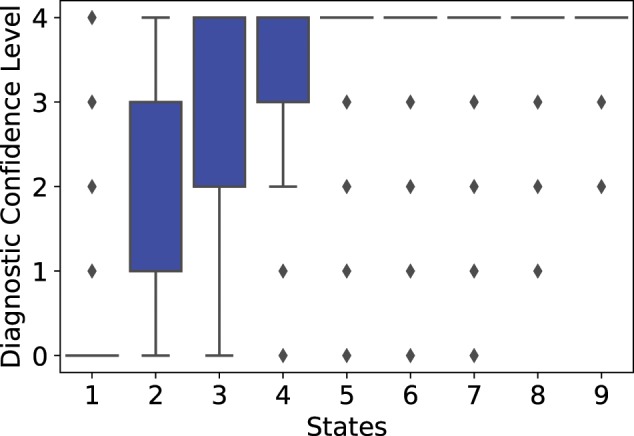
Distributions of diagnostic confidence level at the discovered disease states.

We first checked the distributions of the Diagnostic Confidence Level (DCL) at each discovered state. DCL is one of the most widely used clinical assessment in HD clinical practice. DCL serves as the criterion to determine motor onset (ie, clinical diagnosis) in current clinical practice, despite the fact that these values can be subjective and suffer from personal biases. The value of DCL ranges from 0 to 4. The time a patient’s DCL level first reaches 4 is regarded as the time of motor onset. Figure 2 shows the boxplots of DCL in the nine disease states. According to the distributions of DCL, the nine states can be separated into three phases. In states 1 and 2, most patients had not reached motor onset (DCL <4). Therefore, they are referred to as the *Prodromal* states. The majority of patients went through motor onset during states 3 to 5, so we refer to these as the *Transition* states. By the time patients reached state 6, most patients had already reached motor onset. Therefore, states 6 to 9 are referred to as the *Manifest* states. Comparing to the Shoulson and Fahn stages, IHDPM presents a method to quantify subtle but significant clinical changes in HDGECs well before motor onset is recorded. It covers periods both before and after motor diagnosis, while the Shoulson and Fahn stages only covers periods after motor diagnosis. Furthermore, IHDPM gives a holistic view of the symptom progression in multiple domains.

**Figure 3.**
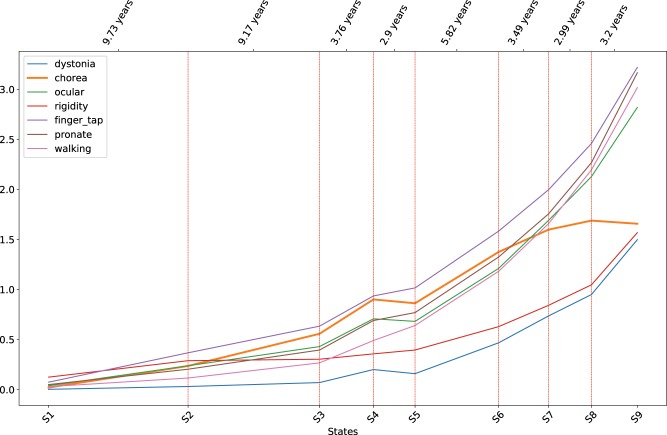
Distributions of motor assessments at the discovered disease states.

**Figure 4.**
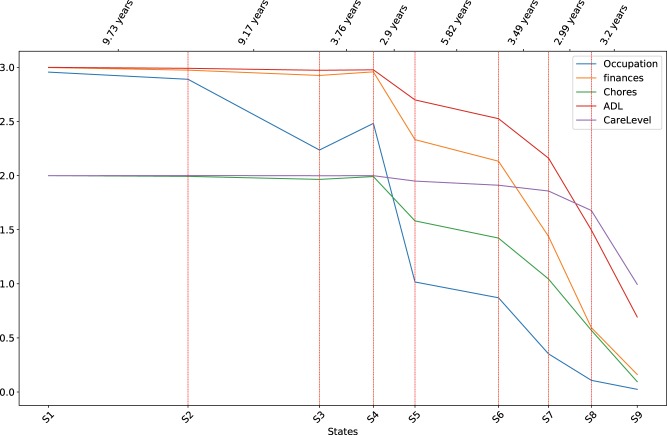
Distributions of functional assessments at the discovered disease states.

**Figure 5.**
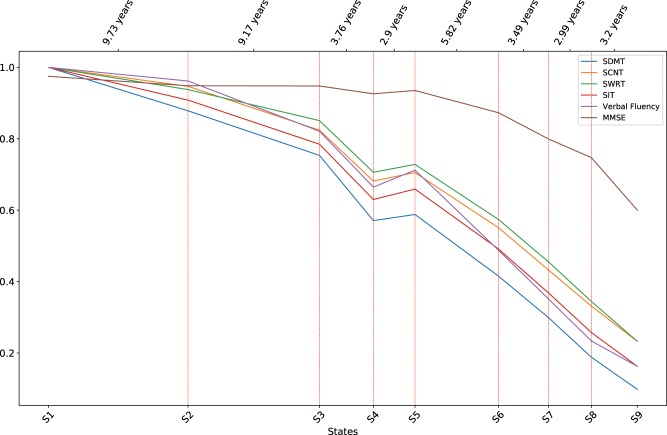
Distributions of cognitive assessments at the discovered disease states.

Figures 3– 5 show the mean values of a selected set of motor, functional, and cognitive assessments at each disease state. Error bars are not shown for cleaner presentations. The distances between disease states on the x-axis are proportional to the expected durations of the states calculated from the transition densities in the model (Table 1). Figure 3 shows that motor scores in general increase with the progression of HD, and Figures 4 and 5 show that functional and cognitive scores decrease with the progression of HD. Note that higher motor scores indicate more severe motor impairment, whereas higher functional and cognitive scores indicate better abilities. The trends in the three plots show that conditions in all three domains in general deteriorate with the progression of HD.

**Table 1.** Expected duration of state 1–8 population

State	1	2	3	4	5	6	7	8
Expected duration (years)	9.7	9.2	3.8	2.9	5.8	3.5	3.0	3.2

The motor and cognitive scores start to deteriorate from the *Prodromal* period. Most functional scores stay relatively stable in early states, except that the Occupation score starts to change as early as state 2, indicating that the inability to work in an employed HD population is an early indicator of functional impairment in HD.

For the *Transition* period, the plots indicate that states 3 and 4 can be distinguished by motor and cognitive changes, with minor changes in the functional domain. On the other hand, states 4 and 5 are distinguished by the sharp drop in functional scores, while motor and cognitive scores do not show significant differences. Recall that previous HD clinical studies and the Shoulson and Fahn stages treat motor diagnosis as the benchmark event in HD progression, and rely on the subjective measure DCL to identify motor diagnosis. IHDPM revealed that patients may undergo complex changes around the time of motor diagnosis.

During the *Manifest* period, among the motor scores, changes in the chorea score (green bold line in Figure 3) deviates from others. The chorea score reaches its peak at state 7 and stays relatively stable afterwards. This observation is congruent with clinical practice where motor diagnosis is contingent upon clear and unambiguous signs of chorea. The subsequent attenuation observed in chorea scores beyond state 7 is also in line with the current understanding that beyond motor diagnosis chorea becomes less pronounced (http://web.stanford.edu/group/hopes/cgi-bin/hopes_test/motor-symptoms/#late-stages). In the cognitive domain, the Mini-Mental State Exam score stays relatively stable until late in the *Manifest* period. The finding is consistent with the knowledge that dementia is not a prominent symptom in early stages of HD.^23^

**Figure 6.**
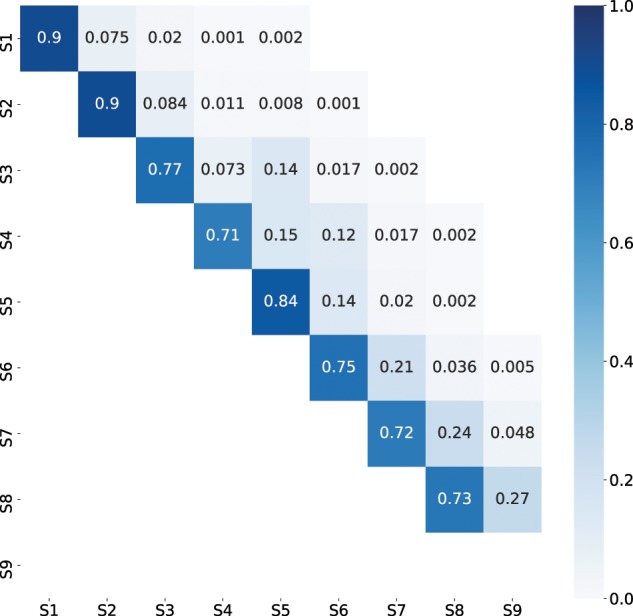
Heatmap of transition probability matrix A(1).

Next, we examine the transitions between the discovered disease states. Recall that the matrix Q represents instantaneous transition rates, and the transition probability matrix over time duration δ can be calculated by equation (1). In this section, we present the transition probability matrix for δ equals to 1 year and denote it as A(1). Figure 6 shows the heatmap of A(1). The (i,j)th element in A(1) represents the probability that a participant at state i ends up at state j at the end of 1 year.

Recall that we assumed a second-order forward-chain progression for HD. Consequently, all elements in the lower triangle of A(1) equal to 0, and they are not marked in Figure 6. Elements on the diagonal represents the probabilities of staying in the same state after 1 year, and the upper off-diagonal elements represent the probabilities of moving to a later state after 1 year. Elements on the diagonal line are generally larger than the off-diagonal elements, indicating that the majority of participants would stay in the same state at the end of 1 year, and only a small portion of participants would move to a more advanced disease state. The observation is consistent with the knowledge that HD has a long duration. Notice that the cells (i,j) with j>i+1 represent the probabilities of “skipping” one or more disease states, and ending up in more advanced states at the end of 1 year. Figure 6 demonstrates that most skipping probabilities are insignificant except during the *Transition* period, that is, cells (3,5) and (4,6). Together with the observations in the previous paragraph, the two nonignorable skipping probabilities suggested that states 4 and 5 could be two parallel states, and there could be multiple potential progression pathways during the transition period. Further investigation of subcohorts with different progression pathways will be a focus of our future work.

Another advantage of IHDPM compared to the Shoulson and Fahn stages is that the expected time durations of the disease states can be calculated. The expected time durations (measured in years) of states 1–8 are summarized in Table 1. No estimated duration for the last state is available since it was set to be an absorbing state. IHDPM infers disease states from HD observations datasets that cumulatively track approximately four decades in HD progression pathway. The expected duration of the *Prodromal* period as defined by these datasets is close to 20 years. It is consistent with the knowledge that subtle changes could happen to patients long before motor onset. The expected duration of states 6–8 together is about 10 years. The observation is consistent with the previous literature that the life expectancy of HD is around 10–15 years after motor diagnosis.^24^

### Individual disease staging

Next, we examine the state sequences of individual patients and compare with the Shoulson and Fahn stages. Table 2 shows an example of a real patient in the integrated HD data. The three columns are the dates of study visits aligned by the first visit (year), the states under the IHDPM model, and the Shoulson and Fahn stages. Note that the Shoulson and Fahn stage only covers period after motor onset, we set visits before motor onset as “premanifest” stage. The patient had a total of 14 visits. According to the Shoulson and Fahn stages, the first 8 visits are in the “premanifest” stage. However, the IHDPM model shows the more granular progression from state 2 (Prodromal) to state 3 (Transition). In the 7th and 8th visits, the patient moved to state 3, indicating that he was getting closer to motor onset. The patient reached motor onset at the 9th visit, and stayed in HD1 until the 13th visit. The IHDPM model shows more detailed progression from state 3 to 5 during this period. In the last visit, the patient moved to HD2 under the Shoulson and Fahn stages, and the IHDPM model also shows the progression from state 5 to 6. The example demonstrates that the IHDPM model provides a more nuanced view about patients’ condition and progression, and can be used to identify patients who are about to enter motor onset or stages. Such information can help with personalized care management, and could be used as a criterion for subcohort segmentation and patient recruitment in clinical trials.

**Table 2.** State sequence of an example patient

Visit date (years)	State from IHDPM	Shoulson and Fahn Stage
0	2	Premanifest
1.1	2	Premanifest
2.1	2	Premanifest
3.5	2	Premanifest
4.2	2	Premanifest
5.7	2	Premanifest
6.6	3	Premanifest
7.5	3	Premanifest
8.9	3	HD1
10.3	4	HD1
11.4	4	HD1
11.8	4	HD1
13.6	5	HD1
14.7	6	HD2

## DISCUSSION AND CONCLUSION

We describe a framework to build disease progression models based on observational data. The method was applied to an integrated observational HD dataset to inform a HD progression model. The learned disease progression model could 1) provide comprehensive view of disease states across the entire progression pathway that is covered by the data; 2) characterize progression of disease as the transition between disease states; 3) generate expected durations of disease states for a targeted cohort; 4) infer disease state sequences for individual patients.

The framework is not limited to HD and could be applied to observational data of other diseases. However, care should be taken when applying the framework to other diseases. For instance, we build a second-degree forward progressing model for HD based on the knowledge that HD has long progression and has no known treatment, and the relevant clinical domains were determined based on data availability and existing knowledge of HD symptoms. Such choices need to be made with the help of clinical knowledge when applying the framework to other diseases.

Quantitatively evaluating the performance of developed HD progression model is difficult due to the lack of a proper gold standard. For example, the state-of-art Shoulson and Fahn stages only cover the postmotor diagnosis period, and relies solely on functional assessments as the criterion to separate the stages. From the discussion of Figures 3–5, we demonstrated that the Shoulson and Fahn stages cannot sufficiently serve as a gold standard for evaluating disease course before motor diagnosis. Other widely used clinical assessments, such as DCL, suffer from biases and noises, and do not serve as appropriate gold standards. In this study, we use existing knowledge in the HD clinical literature (eg, life expectancy) and domain experts’ feedback (eg, observed pattern in chorea score in the Manifest period) to qualitatively validate the developed model. Validating the developed model using independent datasets as well as translating it into clinical practice will be one of the areas of focus in our future research.

## SUPPLEMENTARY MATERIAL

Supplementary material is available at *Journal of the American Medical Informatics Association* online.

## CONTRIBUTORS

Z.S. constructed the dataset, designed the method, and conducted the analysis; G.S., Y.C., and J.H. helped design the method and conceive the project; Y.L. helped construct the dataset; A.M. and S.C guided clinical questions and helped conceive the project; all reviewed the manuscript.

## Supplementary Material

Supplementary Data
