# Supplementary material for: A probabilistic disease progression modeling approach and its application to integrated Huntington’s disease observational data
Source: JAMIA Open. 2019 Jan 7;2(1):123–30. doi: 10.1093/jamiaopen/ooy060 (PMC6951948; doi:10.1093/jamiaopen/ooy060)

**Supplementary Materials**

**S1: Datasets**

In this study, we used an integrated data from four large-scale prospective observational studies of HD, which are named Enroll-HD[17], REGISTRY[18], TRACK-HD/TRACK-ON[13, 19], and PREDICT-HD[20], respectively.

Enroll-HD is a worldwide observational study of HD families. The study monitors how HD appears and changes over time in different subjects, and is open to confirmed HD patients, HD at-risk patients, HD genotype negative participants, as well as control participants from an HD family. Study participants are required to visit study sites annually, and undergo a comprehensive battery of clinical assessments. In this study, we used the ENROLL-IDS- 2015-10-R1 version of the Enroll-HD periodic data, which contains un-monitored data from 7614 subjects who made their baseline visits prior to October 2015. Among the participants, 5475 are HDGECs with CAG length greater than 35, 1613 participants are control subjects with CAG length less than or equal to 35, and the other 527 have unknown CAG length. Subjects have up to four annual visits, with an average number of visits being 1.44.

REGISTRY is a multi-center, multi-national observational study, managed by the European Huntington’s Disease Network (EHDN), with no experimental intervention. REGISTRY aims at obtaining natural history data on many HD mutation carriers and individuals who are part of an HD family, relating phenotypic characteristics of HD, expediting the identification and recruitment of participants for clinical trials, developing and validating sensitive and reliable outcome measure for detecting onset and change over the natural course of pre-manifest and manifest HD. The REGISTRY cohort used in this study consists of 12108 participants, among which 7988 are HDGECs (i.e. CAG > 35), 758 are control participants (i.e. CAG length ≤ 35), and the other 3894 participants do not have CAG length information. Participants have up to 15 annual visits, with an average of 2.9 visits.

TRACK-HD is a multinational study of HD that examines clinical and biological findings of disease progression in individuals with pre-manifest HD and early-stage HD. Participants in the study underwent annual clinical assessments for 36 months. At the baseline visit, 402 participants were enrolled. Among the participants, 127 participants were control subjects, 144 participants were pre-manifest subjects who had not reached HD clinical onset, and 130 were post-manifest subjects who had already reached HD clinical onset. 298 participants completed the 36-month follow- up, among which 97 were controls, 104 were pre-manifest subjects at their baseline visits, and 97 were post-manifest subjects at their baseline visits.

TRACK-ON is a follow-up study of TRACK-HD with the aim of testing for the compensatory brain networks after structural brain changes in TRACK-HD pre-manifest participants. Participants in the study underwent annual clinical assessment for 24 months. At the baseline visit, 245 participants were enrolled, among them 181 were participants of TRACK-HD who have not reached HD clinical onset at the end of TRACK-HD, and 64 participants were newly recruited in the study. 112 participants in TRACK-ON were control subjects, and others are HDGECs.

PREDICT-HD is another longitudinal observational study of subjects who chose to undergo predictive testing for the CAG expansion in the HD gene but did not meet criteria for a diagnosis of HD (Diagnostic Confidence Level = 4). Participants were recruited from 32 sites in the United States, Canada, Australia, and Europe beginning in October 2002. The goal of PREDICT-HD is to define the neurobiology of Huntington’s disease (HD) and to develop tools to allow clinical trials of potential disease-modifying therapies before at-risk individuals have diagnosable symptoms of the disease. It collected a variety of biosamples including MRI, blood and urine samples, and comprehensive assessments of cognitive, motor, functional and psychiatric outcomes to characterize the pre-manifest syndrome in HD, to document the rate of change of these variables during the years leading up to and following a clinical diagnosis of HD, and to investigate the relationship among neurobiologic factors, clinical diagnosis and CAG repeat length. The PREDICT-HD data used in this study consists of 1,481 participants. Among them 316 were control subjects. Participants have up to 14 annual study visits, with an average of 5.2 visits.

**S2: Data Integration Steps**

To integrate data from the four studies, we began by matching subjects across studies using a unique Recoded HD participant ID. This unique identifier also allows us to recognize the subjects who participated in multiple studies. In the four HD observational studies, participants visited study sites approximately annually and were evaluated by a diverse range of clinical assessments. In the rest of this paper, we refer to the data generated from one visit of one participant as an observation. In each of the four studies, the date of a participant’s first visit in the study, referred to as the baseline visit, was used as the reference date for the participant and was set to 0. The visit dates of all his follow-up visits in the same study were aligned with the reference date and measured in days. In addition, for a subject who participated in multiple studies, the time gaps between the multiple reference dates from different studies were also available. Therefore, subjects’ records from multiple studies could be stitched together when they were available.

The second step of combining the multiple data sets was matching and merging variables. Not all variables were named consistently across studies. We analyzed data dictionaries, study protocols and guidelines from the four studies and manually matched variables across studies. We also corrected coding inconsistencies across studies.

We categorized variables into two groups, namely, assessment score and demographical information. The assessment score group consists of measurements from clinical assessments performed at annual study visits to capture wide range of clinical symptoms among HDGECs, such as motor impairment, cognitive deficits, functional decline, and behavioral disorder. The demographic information group includes participants’ demographics (e.g. age, sex, education level, etc.), CAG length, medical history (e.g. drug abuse history, alcohol abuse history, etc.), and other information related to study designs (e.g. region, study site). The integrated data set contains 106 variables from the participants demographic information group and 2,079 variables from the assessment scores group.

Next, we manually compared the range of observed values of each clinical assessment with the data dictionary and study guidelines. Values outside the valid range are regarded as errors and are discarded.

After the above data integration steps, we ended up with a data set containing 55782 observations from 16553 HDGECs and 2716 control participants. The average number of observations per participant of the integrated data is 2.9. Figure S1 shows the distribution of different categories among all observations. Figure S2 shows the histogram of the number of visits (observation length) per patients. 4617 HDGECs have at least 4 visits, and among them 3126 do not have missing values in the clinical features involved in the Robust Latent Variable Analysis[22]. Figure S3 shows the distribution of studies among all observations. Figure S4 shows the distribution of region among all participants. Figure S5 shows the distribution of baseline age among all participants. The red vertical line marks the CAG length of 35, which is the threshold value to distinguish HDGECs and control participants.

**S3: Individual State Sequences**

Once the disease progression model is built, we can obtain the state sequence for any patient, regardless of the number of observations (i.e. study visits) available for individual patients. For patients with only cross-sectional information (i.e. only one study visit), the likelihood value in the observational model (i.e. $P(Z_{n,t}|S=s)$) serves as the criterion for determine the disease state. The state with the highest value of the likelihood is determined as the optimal state. For patients with longitudinal information (i.e. at least two study visits), the standard Viterbi algorithm is used to generate the optimal state sequences $S^{*}=\left\{ S_{1}, S_{2}, \ldots, S_{T} \right\}$, s.t.

$S^{*}=arg\max_{S} P\left( S \right|Z)$.

**S4: Prediction of Future Disease State and Feature Values**

For a patient with $T$ study visits, the observed feature values are denoted as $Z= \left\{ Z_{1}, Z_{2}, . . .,Z_{T} \right\}$ we would like to get the prediction of future disease state, denoted as $S(\delta)$, and future feature values, denoted as $Z(\delta)$, where $\delta$ denotes the time duration after the last visit. We use the intermediate results from the disease progression model. Specifically, we use the forward-backward algorithm and calculate the posterior probability of visit $T$ belonging to disease state $s$ give the observed sequence $Z$, i.e. $P\left( S_{T}=s \right|Z)$, for $S=1, \ldots, M$. Next, the posterior probability of $S\left( \delta\right)=s$ can be calculated as follows:

$P\left( S\left( \delta\right)=s \right|Z)=\sum_{m=1}^{M} P\left( S_{T}=m \right|Z) A_{m,s}(\delta)$.

The state with the highest posterior probability is regarded as the optimal disease state $\delta$ after the last visit. Given the parameters in the observational model $\mu_{m,k}$, the future feature value $Z_{\delta,k}$ can be calculated as follows:

$Z_{\delta,k}=\sum_{m=1}^{M} P\left( S\left( \delta\right)=m \right| Z)\mu_{m,k}$ .

Table S1. Percentage of variations explained by the extracted latent factors

|  | Motor | Functional | Cognitive |
| --- | --- | --- | --- |
| Factor 1 | 60 | 85 | 87 |
| Factor 2 | 11 | 10 | 7 |
| Factor 3 | 6 | 4 | 4 |

Figure S1. Distribution of categories among all observations


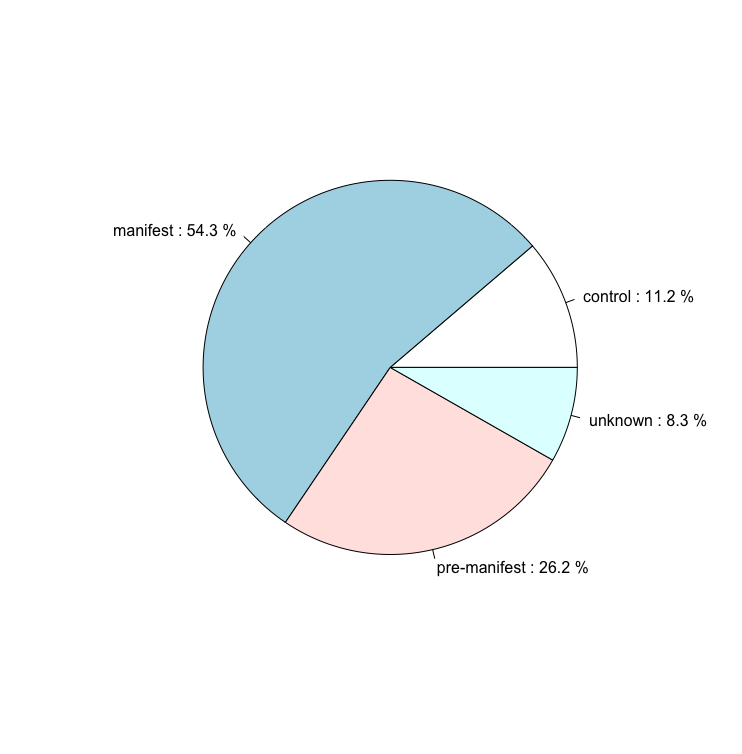


Figure S2. Histogram of the number of study visits per participants


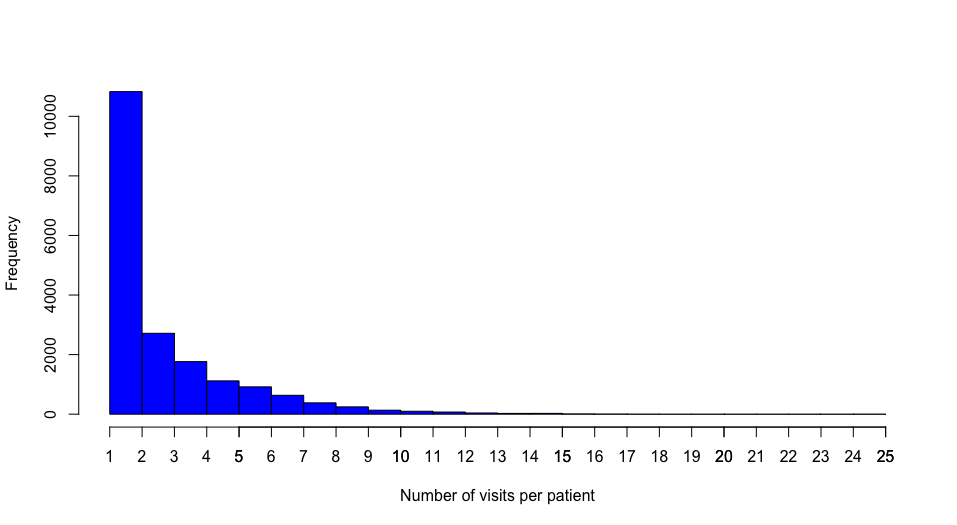


Figure S3. Proportions of Studies among all observations


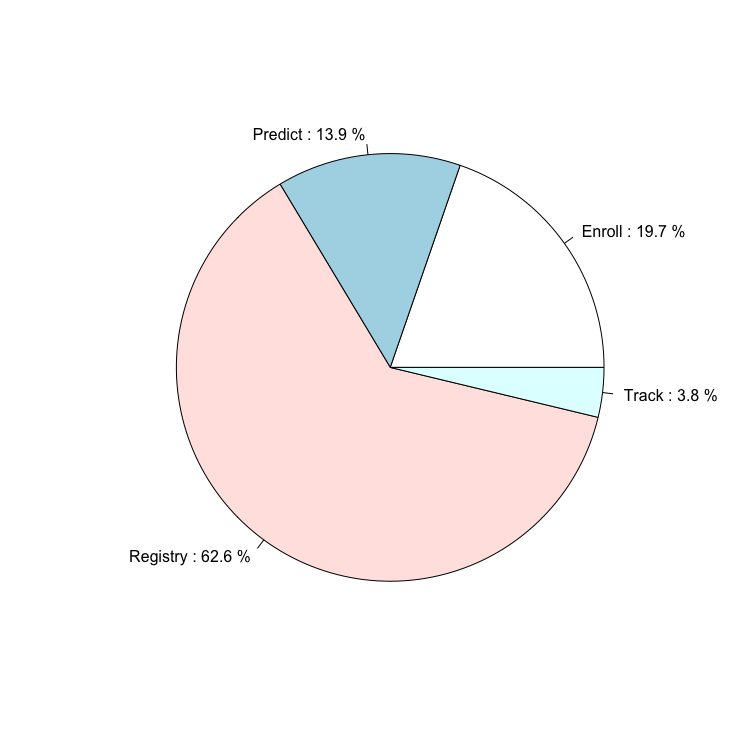


Figure S4. Distribution of region among all participants


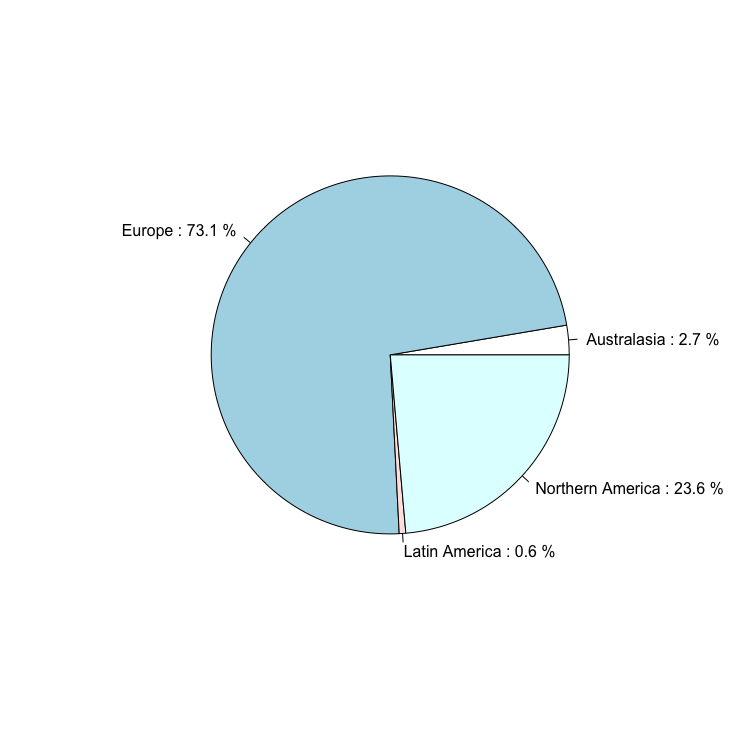


Figure S5. Distribution of age at baseline visit among all participants


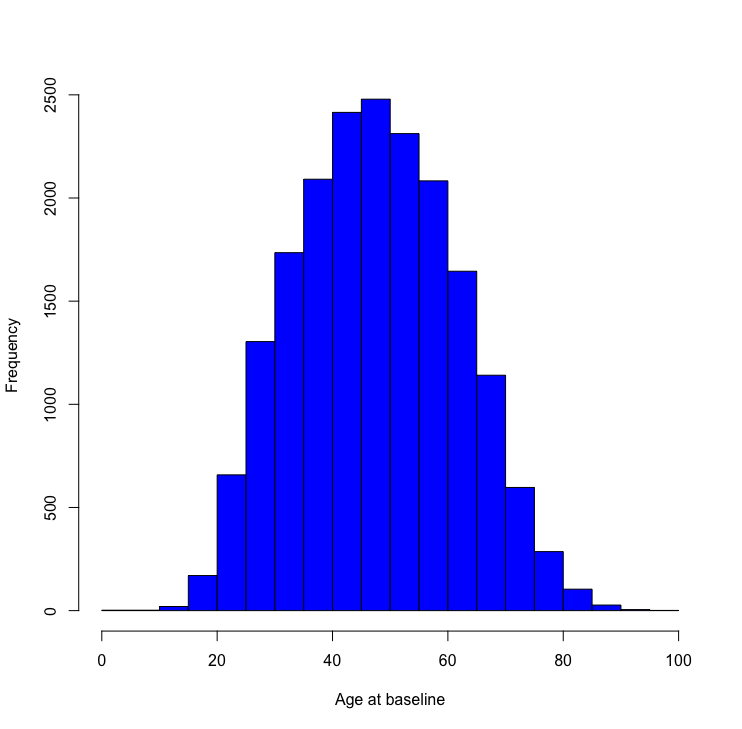


Figure S6. Distribution of CAG repeat length among all participants


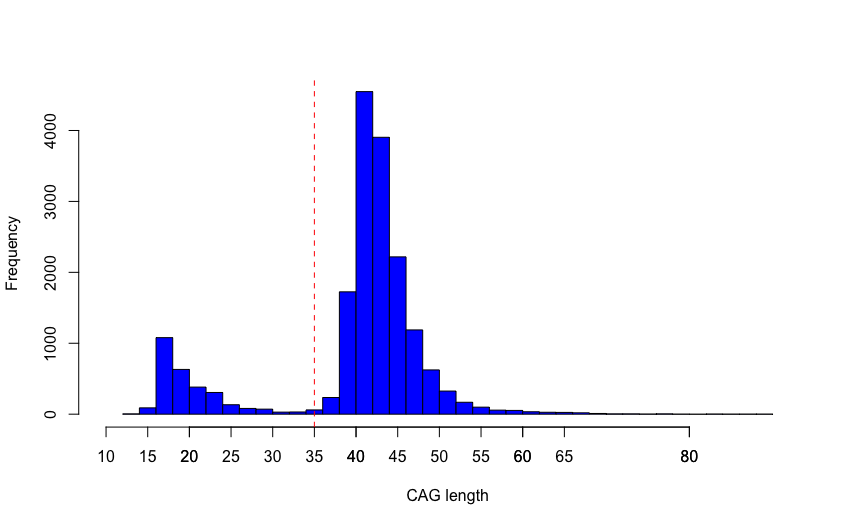

Supplement: Supplementary Data [file ooy060_supp.docx]
